# Providing freedom or financial remuneration? A cross-sectional study on the role of monetary and legal incentives on COVID-19 further booster vaccination intention in the Italian context

**DOI:** 10.3389/fpubh.2023.1186429

**Published:** 2023-06-20

**Authors:** Serena Barello, Marta Acampora, Michele Paleologo, Lorenzo Palamenghi, Guendalina Graffigna

**Affiliations:** ^1^EngageMinds HUB—Consumer, Food and Health Engagement Research Center, Università Cattolica del Sacro Cuore, Milan, Italy; ^2^Department of Psychology, Università Cattolica del Sacro Cuore, Milan, Italy; ^3^Faculty of Psychology, Università Cattolica del Sacro Cuore, Milan, Italy; ^4^Faculty of Agriculture, Food and Environmental Sciences, Università Cattolica del Sacro Cuore, Cremona, Italy

**Keywords:** booster vaccination, COVID-19 vaccine, monetary incentive, legal incentives, vaccination intention, public health policy

## Abstract

Vaccine hesitancy became a more and more important issue during the COVID-19 pandemic. Due to the emergence of new variants, many international health agencies have already begun administering booster doses of the vaccine in response to these threats. Studies have emphasized the effectiveness of different types of incentive-based strategies to increase vaccination behaviors. The purpose of the present study was to identify the correlation between different types of incentives (legal or financial) with people’s intentions to get a COVID-19 booster vaccine. We conducted a cross-sectional study between 29 January 2022 and 03 February 2022. An online quantitative survey was carried out in Italy. One thousand and twenty-two Italian adults were recruited by a professional panel provider. Descriptive statistics were computed for the five variables concerning the incentives (monetary, tax, fee, health certification, travel) toward vaccination. A general linear model (GLM) was then computed to compare the scores of the five different variables within the subjects. The general linear model showed a significant within-subjects main effect. Post-hoc comparisons showed that among the financial incentive, the monetary reward is rated lower than all the others. Tax and fees both resulted lower than both the legal incentives. Finally, COVID-19 health certification and travel did not result significantly different from each other. This study offers an important contribution to public policy literature and to policymakers in their efforts to explain and steer booster vaccination acceptance while facing an ongoing pandemic.

## 1. Introduction

Vaccine hesitancy, has become an increasingly important issue during the COVID-19 pandemic, to the extent that it was identified in 2019 by the World Health Organisation (1) as a major threat to global health. Due to the emergence of new variants, many international health agencies have already begun administering booster doses of the vaccine in response to these threats.

If vaccine acceptance has been a problem since the beginning of the pandemic, the administration of future booster shots could increase the hesitancy phenomenon, as studies have shown (2–8). As of 21st, July 2022 (at the time of writing this manuscript), only 107 million fully vaccinated people worldwide have received an additional vaccine dose or a booster dose, the highest level of protection against the virus.

Given these figures, the phenomenon of vaccine hesitancy will continue to be a serious threat to the end of the COVID-19 pandemic and for this reason it is necessary to investigate the mechanisms underlying this phenomenon by taking opportunity of large-scale vaccination due to the recent health emergency as a field of study.

Low vaccination intentions have been linked to people’s lack of trust in the safety of vaccines, complacency (seeing vaccination as largely unnecessary), calculativeness (carefully weighing risks and benefits), obstacles to vaccination, and low collective responsibility (e.g., unwillingness to get vaccinated to protect others) perceptions, according to previous research involving healthcare workers and the general population (9–12). Researchers have suggested a range of interventions, from informational campaigns to mandatory vaccination, for addressing these vaccine antecedents and boosting vaccination intentions (13–15). Offering incentives for vaccination could increase vaccination intentions in the same way that incentives have been demonstrated to encourage other healthy habits, such as keeping a healthier diet, stopping smoking, or doing physical exercise (16–18).

The literature (19–22) on this topic has emphasized the effectiveness of different types of incentive-based strategies to increase vaccination behaviours. Several studies (23–26) have shown how incentive-based strategies based on financial remuneration (e.g., monetary, bonus) in different countries have increased the acceptance of vaccines. Other studies (27–32) have also shown that the use of legal incentives linked to providing freedoms (e.g., the possibility to travel, the possibility to participate in public activities) are effective in promoting vaccination campaigns. However, vaccine hesitancy persists among certain population segments, necessitating further research into effective strategies for addressing this issue (33).

In this scenario, it is crucial to understand the effective approaches that can motivate the hesitant population to receive uptake doses of the COVID-19 vaccine, leveraging this health emergency as a field of study to gain a deeper understanding of vaccine hesitancy as a whole. Indeed, despite certain unique aspects related to COVID-19 (15), being against vaccine remains a significant barrier to COVID-19 vaccination (34). Therefore, effective strategies in this specific context may also prove effective in the future, presenting an opportunity to bridge the gap between scientific potential and citizen behavior.

Based on these premises, the purpose of the present study was to identify the correlation between different types of incentives (legal or financial) with people’s intentions to get a COVID-19 booster vaccine.

## 2. Methods

### 2.1. Sample and procedure

One thousand and twenty-two Italian adults were recruited by a professional panel provider (Norstat Italia Srl) by employing a stratified sampling. After providing their informed consent, the participants were asked to fill an online survey (using a CAWI methodology). The survey included questions regarding the participants’ sociodemographic status (gender, age, monthly family wage, level of education); one question regarding their COVID-19 vaccinal status, namely whether they did the booster dose, scheduled it, or did not do it nor scheduled it; and five questions regarding their intention to do an additional anti-COVID-19 vaccinal dose if an incentive were provided. Incentives were either financial (monetary, tax relief, or a fee in case of non-compliance), or legal (COVID-19 health certification, or freedom to travel). Participants were asked to rate their agreement on a 6 steps Likert scale (1 = strongly disagree, 6 = strongly agree).

Participants that did not do, nor scheduled the booster dose were excluded from the sample; the same goes for participants who refused to answer the question regarding their monthly wage.

### 2.2. Statistical analyses

First, frequencies were calculated for the sociodemographic characteristics of the sample. Descriptive statistics (mean, standard deviation, skewness and kurtosis) were computed for the five variables concerning the incentives towards vaccination. The scores for the five variables were also transformed in *z*-scores and screened for outliers (*z* ≥ |3|).

A general linear model (GLM) was then computed to compare the scores of the five different variables within the subjects. Gender, wage (coded as above and below the median of 1800€/month), and education (coded as no high school degree, high school degree, and university degree) were also included in the model as between-subjects variables. Interactions with the within-subject variable were also included in the model, but no interactions between the between-subject variables were computed. Mauchly’s test of sphericity was computed to verify the assumption of sphericity, and the appropriate correction was then applied to correct for the violated sphericity, depending on the resulting *ε*: Greenhouse–Geisser correction for <0.75 (35), and Huynh–Feldt correction for *ε* > 0.75 (36). Partial eta-squared (*η*_p_^2^) was calculated as effect size for the *F*-tests. Post-hoc analyses were calculated, using the Holm-Bonferroni correction (37), to inspect pairwise differences between the different levels of the within-subject dependent variable in the overall sample, and -where an interaction resulted significant in the different levels of the independent variables; Cohen’s *d* was calculated as effect size for these comparisons.

All the analyses were run using JASP software v0.16.

### 2.3. Ethical considerations

This study has been performed in accordance with the Declaration of Helsinki and has been approved by an independent ethics commission of the Department of Psychology of Università Cattolica del Sacro Cuore in Milan (CERPS).

## 3. Results

### 3.1. Descriptive statistics

Two hundred and thirty-five participants were removed as they indicated that they did not do the recommended vaccination cycle, nor did they schedule it. Further 113 participants were removed as they showed missing data on the question regarding wage. The overall remaining sample was *N* = 674. The average age in the sample was 48 (SD = 13, range between 20 and 72). Table 1 shows the descriptive statistics of the sample: gender, geographical area of residence, education level, and family monthly wage. Descriptive statistics of the values of the intention of the sample to do an additional dose under the five different incentive type conditions (monetary, tax, fee, health certification and travel) were also conducted. As shown in Table 2, the results show that legal incentives are more endorsed than financial incentives by respondents. The screening of the outliers based on the *z*-scores showed that no outliers were present in the sample.

**Table 1:** Sample characteristics.

Variables	*n*	%
Gender		
Male	330	48.96
Female	344	51.04
Geographical area of residence		
North-west	177	26.26
North-east	121	17.95
Center	122	18.10
South & islands	254	37.69
Education level		
No degrees	1	0.15
Elementary degree	2	0.30
Middle school degree	106	15.73
High school degree	375	55.64
University degree	190	28.19
Family monthly wage		
Up to 600€	37	5.49
601–900€	38	5.64
901–1,200€	69	10.24
1,201–1,500€	92	13.65
1,501–1800€	64	6.50
1801–2,500€	138	20.48
2,501–3,500€	126	18.69
3,501–4,500€	72	10.68
More than 4,500€	38	5.64

**Table 2:** Descriptive statistics of the values of the intention to do an additional dose under the five different conditions.

I will do further doses of vaccine, in addition to the current “booster” dose, if *(item text)*…	Label of the variable	Type of incentive	Mean	Std. dev.	Skewness	Kurtosis
… I will receive a monetary reward for vaccinating	Monetary	Financial	3.095	1.802	0.275	−1.309
… the government will reduce my taxes	Tax	Financial	3.562	1.721	−0.097	−1.236
… the government will fee me for not vaccinating	Fee	Financial	3.635	1.619	−0.197	−1.019
… I will receive a COVID-19 health certification (HC) that will allow to avoid limitations to my daily life	HC	Legal	4.352	1.383	−0.736	−0.060
… this will allow me to travel freely	Travel	Legal	4.282	1.480	−0.658	−0.364

### 3.2. General linear model

Mauchly’s test of sphericity resulted significant [*χ*^2^(9) = 326.230; *p* < 0.001; *ε* = 0.805]: Huyn–Feldt correction was then applied for the subsequent analyses.

The general linear model showed a significant within-subjects main effect [*F*(3.220, 2154.115) = 117.115; *p* < 0.001; *η*_p_^2^ = 0.149]. Post-hoc comparisons (see Table 3) show that among the financial incentive, the monetary reward is rated lower than all the others with *p* < 0.001. Tax and Fee did not result significantly different from each other (with *p* = 0.396), but both resulted lower than both the legal incentives (i.e., health certification and travel) with p < 0.001. Finally, HC and Travel did not result significantly different from each other (with *p* = 0.307).

**Table 3:** Post-hoc comparisons of the different incentives.

		Mean difference	95% CI for mean difference		SE	*t*	Cohen’s *d*
			Lower	Upper			
Monetary	Tax^b^	−0.456	−0.652	−0.260	0.070	−6.539^a^	−0.252
	Fee^c^	−0.515	−0.711	−0.319	0.070	−7.388^a^	−0.285
	HC^d^	−1.275	−1.471	−1.079	0.070	−18.283^a^	−0.704
	Travel^e^	−1.175	−1.371	−0.979	0.070	−16.855^a^	−0.649
Tax	Fee^c^	−0.059	−0.255	0.137	0.070	−0.849	
	HC^d^	−0.819	−1.015	−0.623	0.070	−11.744^a^	−0.452
	Travel^e^	−0.719	−0.915	−0.523	0.070	−10.316^a^	−0.397
Fee	HC^d^	−0.760	−0.956	−0.564	0.070	−10.895^a^	−0.420
	Travel^e^	−0.660	−0.856	−0.464	0.070	−9.467^a^	−0.365
HC	Travel^e^	0.100	−0.096	0.295	0.070	1.428	

Results are averaged over the levels of: gender, wage, education.

a Significative (*p*_holm_ < 0.001).

b The government will reduce my taxes.

c The government will fee me for not vaccinating.

d I will receive a COVID-19 health certification (HC) that will allow to avoid limitations to my daily life.

e This will allow me to travel freely.

Additionally, a between-subjects main effect of the gender variable resulted significant [*F*(1, 669) = 8.647; *p* = 0.003; *η*_p_^2^ = 0.013], with males having an overall mean of 0.283 (95% CI, 0.094, 0.472) above females. No significant main effect for wage (*p* = 0.971) and education (*p* = 0.637) emerged from analyses.

Finally, a marginally significant interaction gender × incentives resulted from analyses [*F*(3.220, 2154.115) = 4.368; *p* = 0.004; *η*_p_^2^ = 0.006]. The post-hoc analyses (see Table 4) showed that there is a significant difference in the mean of the monetary incentive between the male and the female group, with males having a higher mean answer than female with *p* < 0.001. No other comparison resulted significant.

**Table 4:** Post-hoc comparisons of the gender × incentives interaction.

	Mean difference (male – female)	95% CI for mean difference		SE	*t*	Cohen’s *d*	*p*_holm_
		Lower	Upper				
Monetary^e^	0.572	0.167	0.977	0.124	4.617	0.309	<0.001
Tax^a^	0.346	−0.059	0.751	0.124	2.792		0.074
Fee^b^	0.121	−0.284	0.526	0.124	0.979		1.000
HC^c^	0.163	−0.242	0.568	0.124	1.318		1.000
Travel^d^	0.213	−0.192	0.618	0.124	1.715		0.865

Only the meaningful comparisons are shown in this table. Results are averaged over the levels of: wage, education.

a The government will reduce my taxes.

b The government will fee me for not vaccinating.

c I will receive a COVID-19 health certification (HC) that will allow to avoid limitations to my daily life.

d This will allow me to travel freely.

e I will receive a monetary reward for vaccinating.

A marginally significant effect was also noted in the wage × incentives interaction [*F*(3.220, 2154.115) = 3.866; *p* = 0.007; *η*_p_^2^ = 0.006]; however, post-hoc analyses showed no particular differences of interest of the dependent variables between the groups of people with higher or lower wages.

## 4. Discussion

In this study, we investigated the effects of legal and financial incentives on COVID-19 booster dose vaccination intentions. Our results indicated that incentives are a suitable mean to motivate citizens to increase their willingness to get vaccinated even in the case of booster doses. Furthermore, we discovered that both types of incentives significantly relate with peoples’ willingness to vaccinate against COVID-19. However, for our sample, legal incentives—and in particular the introduction of vaccination health certificates required to access specific venues and being allowed to travel—were reported as the most effective incentive to boost vaccination intentions as indicated by other studies (25, 38, 39).

Our results are also in line with other studies that reported positive impacts of financial incentives on booster vaccination (23, 40, 41). Indeed, while vaccination mandates seem to be more likely to increase primary vaccination, incentives could be implemented to sustain booster uptake (42).

Indeed, as showed by other studies on the role of ethnicity in modifying the relationship between incentives and health behaviour change (43, 44), it is possible that for various populations and cultural backgrounds, the observed impacts of financial and legal incentives would differ. Thus, caution should be used when interpreting our findings. Additionally, several research advice considering the varying effects of rewards on persons with various motivations. According to the psychological literature, a person’s motivation levels may influence how they are influenced by the outside rewards that are given to them to increase their desire to carry out the requested behaviours (45–47). Evidence demonstrates that while typically highly motivated individuals are less influenced by external incentives (48), highly motivated individuals can occasionally be more susceptible to financial incentives than other individuals (49).

This study has some limitations, and results should be interpreted and used with caution. Firstly, the measures used in this study were self-reported and might be subject to reporting bias. In addition, the current study adopted a series of measures that were not validated—even if internal consistency was adequate. Second, as an observational cross-sectional study, causal relationships could not be inferred. Finally, there are indeed some socio-demographic variables that were not considered in this paper, and that is worth discussing. Despite its limitations, this study offers an important contribution to public policy literature and to policy makers in their efforts to explain and steer booster vaccination acceptance while facing an ongoing pandemic. Future research should explore the effects of other monetary and non-monetary types of incentives, as well as the interaction effect of incentive type and valence. Moreover other studies should estimate the impact of compulsory vaccine in acceptance rate and vaccine coverage (50, 51).

## 5. Conclusion

Based on results of this study, policymakers should consider incorporating common incentives into their vaccination promotion campaign, providing monetary incentives, and issuing health certifications—which permit access to public spaces and cultural events. Moreover, the social and cultural context of the intended vaccination target should be considered while designing these incentives.

## Data availability statement

The raw data supporting the conclusions of this article will be made available by the authors, without undue reservation.

## Ethics statement

The studies involving human participants were reviewed and approved by an independent ethics commission of the Department of Psychology of Università Cattolica del Sacro Cuore in Milan (CERPS). The patients/participants provided their written informed consent to participate in this study.

## Author contributions

SB contributed to the conception and design of the study, methodology, and writing original draft. LP performed the statistical analysis. MA contributed to the methodology and preparation writing original draft. MP contributed to the methodology and data curation. GG supervised the research project and contributed to the writing, reviewing, and editing the manuscript. All authors contributed to the article and approved the submitted version.

## Funding

This study was conducted within the CRAFT project, funded by Fondazione Cariplo and Regione Lombardia. The authors declare that the funders had no role in the study design, in interpretation of the results or in the decision to publish the results.

## Conflict of interest

The authors declare that the research was conducted in the absence of any commercial or financial relationships that could be construed as a potential conflict of interest.

## Publisher’s note

All claims expressed in this article are solely those of the authors and do not necessarily represent those of their affiliated organizations, or those of the publisher, the editors and the reviewers. Any product that may be evaluated in this article, or claim that may be made by its manufacturer, is not guaranteed or endorsed by the publisher.
